# Development and validation of a regionally adapted sandwich enzyme-linked immunosorbent assay targeting recombinant p60 antigen for rapid detection of *Listeria monocytogenes* in food samples

**DOI:** 10.14202/vetworld.2025.3839-3854

**Published:** 2025-12-13

**Authors:** Guldarigash Kaukabayeva, Aigerim Turgimbayeva, Zhanar Akhmetkarimova, Sholpan Mukhlis, Gulkhan Unysheva, Sailau Abeldenov, Pavel Shevchenko, Albina Gabitova, Raushan Rychshanova, Aralbek Rsaliyev, Yergali Abduraimov, Saule Eskendirova

**Affiliations:** 1 Stem Cell Laboratory, National Center for Biotechnology, 13/5 Korgalzhyn Highway, Astana, 010000, Kazakhstan; 2 Research Institute of Applied Biotechnology, Baitursynov Kostanay University, Mayakovskiy Street 99/1, Kostanay, 110000, Kazakhstan; 3 National Center for Biotechnology, 13/5 Korgalzhyn Highway, Astana, 010000, Kazakhstan.

**Keywords:** food safety surveillance, foodborne pathogens, hybridoma technology, immunodiagnostics, *Listeria monocytogenes*, monoclonal antibodies, One Health, p60 antigen, polyclonal antibodies, recombinant protein, sandwich enzyme-linked immunosorbent assay

## Abstract

**Background and Aim:**

*Listeria monocytogenes* remains a major foodborne pathogen globally, with mortality rates ranging from 20%–40%. The increasing incidence of listeriosis and the limitations of culture-based and polymerase chain reaction-based diagnostics highlight the need for rapid, cost-effective, and highly specific immunoassays. This study aimed to develop and validate a regionally adapted sandwich enzyme-linked immunosorbent assay (ELISA) based on monoclonal and polyclonal antibodies (pAbs) raised against a recombinant p60 antigen derived from a Kazakhstani *L. monocytogenes* field isolate.

**Materials and Methods:**

The *p60* gene lacking its N-terminal signal peptide was amplified from a regional *L. monocytogenes* isolate, cloned into the pET28c(+) vector, and expressed in *Escherichia coli* arctic express (DE3). Recombinant p60 protein was purified by Ni^2+^-affinity chromatography and used to immunize BALB/c mice and Chinchilla rabbits for monoclonal antibodies and pAbs antibody production. Hybridoma clones were screened for specificity using indirect ELISA and Western blot. A sandwich ELISA was assembled using mAb 1H8 as the capture antibody and horseradish peroxidase-conjugated rabbit pAbs as detection antibodies. Analytical sensitivity and diagnostic performance were evaluated using serial dilutions of recombinant p60 and culture supernatants of *L. monocytogenes* isolates recovered from 507 food samples.

**Results:**

The recombinant p60 antigen (50.3 kDa) was successfully expressed and purified at 5.9 mg/L yield. Among seven stable hybridoma clones, mAb 1H8 exhibited the highest affinity (Ka = 2.5 × 10^10^ M^-1^) and specificity without cross-reactivity to non-Listeria bacteria. The optimized sandwich ELISA achieved a detection limit of 1.5 ng/mL, corresponding to approximately 10^3^ colony-forming units/mL. All six *L. monocytogenes* field isolates tested positive in the assay, with results strongly correlating with viable cell counts (R^2^ = 0.89). The assay demonstrated comparable sensitivity to commercial kits while offering shorter assay time (2 h) and substantially lower production cost.

**Conclusion:**

The developed sandwich ELISA provides a sensitive, specific, rapid, and regionally tailored diagnostic platform for detecting pathogenic *L. monocytogenes* in food samples. By integrating locally produced recombinant antigens and immunoreagents, the assay offers a cost-effective alternative to imported kits, supporting national food safety programs and One Health surveillance initiatives.

## INTRODUCTION

*Listeria monocytogenes* is a major zoonotic pathogen that can infect both humans and animals. Its primary reservoirs include diverse environmental sources and natural substrates. Human infection usually occurs through the alimentary route, most commonly by consuming inadequately cooked or improperly processed food products. Listeriosis is among the most severe foodborne diseases, with reported mortality rates of 20%–40%. The rising incidence of the disease is closely associated with the extended storage of food at low temperatures, which supports the growth of *Listeria* even when initial contamination levels are low [1–3].

Given its high pathogenic potential, rapid and reliable detection of *L. monocytogenes* in raw materials and food products has become a critical public health priority. Early identification in food or environmental samples is essential for effective control measures and prevention of outbreaks. Traditional bacteriological culture methods, requiring selective media, biotyping, and serotyping, are labor-intensive and slow, whereas polymerase chain reaction (PCR)-based assays, though sensitive, remain costly. These limitations underscore the growing relevance of immunological methods for the practical detection of *L. monocytogenes* [4–6].

Immunological assays, such as enzyme-linked immunosorbent assay (ELISA) and lateral-flow immunoassay, offer attractive alternatives due to their low operational cost, high sensitivity and specificity, and ease of result interpretation. Current immunoassay platforms can detect approximately 10^3^–10^5^ colony-forming units (CFU)/mL of whole bacterial cells, whereas recombinant-antigen-based assays can achieve nanogram-level detection limits [4, 5, 7].

*Listeria* is particularly notable for its intracellular lifestyle and exceptional adaptive capabilities [8]. Its virulence is driven by a diverse set of factors that facilitate incomplete phagocytosis, intracellular survival, rapid cell-to-cell spread, and the development of antimicrobial resistance [9, 10]. Key virulence-associated proteins include listeriolysin O, phospholipase C, lecithinase, metalloproteinases, internalins, actin assembly-inducing protein (ActA), and positive regulatory factor A (PrfA), which collectively enable invasion, persistence, and replication within endothelial and epithelial cells of both humans and animals [4, 5, 11, 12].

Among these, the highly conserved p60 protein (80%–90% sequence homology across *Listeria* species) is a prominent cell wall-associated antigen involved in mammalian cell invasion. Encoded by the *iap* gene, p60 functions as a muramidase that hydrolyzes peptidoglycan, thereby facilitating bacterial entry. Its strong immunogenicity and active secretion during cell division make p60 a valuable diagnostic marker and an excellent candidate for detecting *L. monocytogenes* in contaminated food products [12–16].

Despite the availability of several commercial ELISA platforms for *L. monocytogenes* detection, most rely on recombinant antigens and monoclonal antibodies (MAbs) derived from reference strains that may not fully represent regionally circulating genotypes. This mismatch can reduce diagnostic sensitivity in local surveillance settings. In addition, many existing assays require long incubation times, expensive proprietary reagents, and may not offer sufficient analytical performance for low-level contamination typical of refrigerated food products. Importantly, no ELISA system to date has been developed using a recombinant p60 antigen engineered from a Kazakhstan field isolate with strategic deletion of the signal peptide to enhance solubility, stability, and immunoreactivity. Therefore, there is a pressing need for a regionally adapted, cost-effective, and highly sensitive immunoassay that integrates locally produced recombinant antigens and MAbs to improve the rapid detection of *L. monocytogenes* in diverse food matrices.

This study aimed to develop and validate a rapid, sensitive, and regionally tailored sandwich ELISA test system for the detection of pathogenic *L. monocytogenes* in food products. Specifically, the work focused on producing a recombinant p60 antigen from a locally circulating isolate, generating high-affinity monoclonal and polyclonal antibodies (pAbs) against this engineered antigen, and assembling an optimized immunoassay capable of delivering analytical performance comparable to or superior to existing commercial kits. The overarching goal was to create a cost-effective diagnostic platform suitable for routine food safety monitoring and One Health listeriosis surveillance programs.

## MATERIALS AND METHODS

### Ethical approval

The animal procedures performed in this study were conducted in strict accordance with internationally recognized guidelines for the care and use of laboratory animals, including the EU Directive 2010/63/EU, the U.S. National Research Council’s Guide for the Care and Use of Laboratory Animals, and the ARRIVE 2.0 recommendations. All protocols involving BALB/c mice and Chinchilla rabbits used for monoclonal and polyclonal antibody production were reviewed and approved by the Local Ethics Committee of the National Center for Biotechnology Information (NCBI), Republic of Kazakhstan (Protocol No. 1, dated 14 July 2023).

Animals were housed under standard husbandry conditions in accredited NCB animal facilities that comply with Good Laboratory Practice standards and are under continuous veterinary oversight. All procedures, including immunization, blood collection, hybridoma production, and ascitic fluid harvesting, were performed by trained personnel using aseptic techniques to minimize pain and distress. Humane endpoints, environmental enrichment, and routine health monitoring were implemented throughout the study. No unexpected animal morbidity or mortality occurred.

All manipulations involving *L. monocytogenes* were carried out under biosafety level 2 conditions in accordance with ISO 11290, institutional biosafety regulations, and national guidelines for handling pathogenic microorganisms. These procedures included the use of certified biological safety cabinets, appropriate personal protective equipment, validated inactivation protocols, and approved waste-disposal practices. The study did not involve human participants, clinical samples, or field animal sampling; therefore, separate human ethics approval was not required.

### Study period and location

The study was conducted from November 2023 to June 2025 at the National Center for Biotechnology.

### Laboratory animals

Twelve BALB/c mice (1 month old, 45 g live weight) and 3 Chinchilla rabbits (3 months old, 3.2 kg live weight) were used to produce pAb and mAb.

### Cloning, expression, and purification of recombinant p60

The genomic DNA of *L. monocytogenes* (strain ID 1336–1 in the laboratory stock) used in this study was obtained from commercial frozen chicken meat at the Research Institute of Applied Biotechnology, Research and Innovation Center, Non-profit Limited Company “Akhmet Baitursynuly Kostanay Regional University.” This isolate does not have a GenBank accession number because it is not a reference or publicly registered strain.

We isolated genomic DNA from *L. monocytogenes* using a Genomic DNA Purification Kit (Promega, Madison, WI, USA). Before PCR amplification, DNA concentration and purity were verified using NanoDrop (A260/A280 ratio: 58.3 ng/μL, A260/A280: 2.46). The *p60* gene, lacking the N-terminal signal domain, was amplified from the genomic DNA with primers Fw-Nde-Lmp60 (5′-CGCATATGAGCACTGTAGTAGTC-3′) and Rv-BamHI-Lmp60 (5′-ATGGATCCTTATACGCGACCGAAG-3′). PCR was performed as follows: Initial denaturation at 98°C for 2 min; 35 cycles: 98°C–10 s, 55°C–30 s, 72°C–45 s; final elongation at 72°C for 7.5 min. The PCR product was cloned into the pET28c(+) vector at the NdeI-BamHI restriction sites. The sequence of the insert was verified against the GenBank reference sequence using Vector NTI Advance v11.0 software (Invitrogen, USA).

For protein expression, *Escherichia coli* arctic express (DE3) cells were transformed with the pET28c(+)/Lmp60 plasmid. Protein expression was induced in a 1 L culture with 0.5 mM isopropyl β-D-1-thiogalactopyranoside when the culture reached an optical density (OD_600_) of 0.6 at 600 nm (OD_600_). Cells were incubated at +22°C with shaking (20 g) for 16 h and harvested by centrifugation at 6,000 × *g* for 7 min at +4°C. Cell pellets were resuspended in lysis buffer containing 20 mM Tris-HCl (pH 8.0), 150 mM NaCl, and a Complete Protease Inhibitor Cocktail (Roche, Basel, Switzerland).

Cell disruption was performed by treatment with lysozyme (3 mg/mL, 20 min, RT) followed by sonication on ice (pulsed mode, 50 kHz; Ultrasonic Cell Disrupter -1,200, Biobase). The lysate was clarified by centrifugation at 40,000 × *g* for 1 h at +4°C. The supernatant was loaded onto a HisTrap HP 1 mL column (Cytiva, Marlborough, MA, USA), and recombinant protein was eluted using a linear imidazole gradient (50–500 mM). Fractions were analyzed by sodium dodecyl sulfate (SDS)-polyacrylamide gel electrophoresis (12%) to assess purity. Recombinant p60 protein was dialyzed against phosphate-buffered saline (PBS, pH 7.4) at +4°C with multiple buffer exchanges.

### Hybridoma technology

BALB/c mice were immunized intraperitoneally on day 1 with 25–50 μg of recombinant *L. monocytogenes* p60 antigen emulsified in 0.1 mL of incomplete Freund’s adjuvant (IFA) (Thermo Scientific, Massachusetts, USA) in a 1:1 ratio. On day 7, the mice received a second intraperitoneal injection of 25–50 μg of the antigen in an equal volume of IFA. On days 11, 12, and 13, the same booster dose was administered in PBS. Serum samples from immunized mice were tested for antibody production using ELISA 3 days after the final immunization.

Animals with the highest antibody titers were selected, and their spleens were harvested as a source of immune lymphocytes. Hybridization was performed by fusing splenocytes from immunized BALB/c mice with X63–Ag 8.6.5.3 myeloma cells at a 1:10 ratio. Polyethylene glycol 1,500 (50% w/v; Roche Diagnostics, Mannheim, Germany) was used as the fusing agent. Hybrid cell selection was carried out in hypoxanthine–aminopterin–thymidine medium (Sigma-Aldrich, St. Louis, USA).

For cell culture maintenance, RPMI-1640 medium (Gibco, Paisley, UK) supplemented with 10% fetal bovine serum (Gibco) was used. Cells were cultured at 37°C in a 5% CO_2_ atmosphere. Passaging is performed every 3–4 days. Hybrid cell lines were cloned using the limiting dilution method.

### Indirect ELISA

Ninety-six-well plates were coated with recombinant *L. monocyto*genes p60 antigen at a concentration of 1 мкг/mL in 0.1 mL carbonate-bicarbonate buffer (pH 7.2–7.4) and incubated overnight at 4°C. Plates were washed 3 times with PBS containing 0.1% Tween-20 (PBS-T), followed by 3 washes with PBS. Culture supernatants from the investigated hybridoma clones were added to the wells. Control wells contained either culture supernatant from myeloma cells or PBS. Plates were incubated at +37°C for 1 h.

After incubation, the wells were aspirated, washed again as described, and filled with 0.1 mL of a working dilution of horseradish peroxidase (HRP)-conjugated rabbit anti-mouse immunoglobulin antibodies (Sigma-Aldrich, St. Louis) prepared in PBS-T. Plates were incubated at 37°C for 1 h, followed by additional washing steps to remove unbound conjugates. Enzymatic cleavage of the chromogenic substrate 3,3′,5,5′-tetramethylbenzidine (TMB) (Thermo Scientific) was used to detect specific antibodies. The absorbance values were measured at 450 nm using a spectrophotometer.

### Ascitic fluid production and monoclonal antibody purification

Ascitic fluids containing MAbs against recombinant *L. monocytogenes* p60 protein were obtained by propagating hybridoma cells in the peritoneal cavity of syngeneic mice pretreated with 2,6,10,14-tetramethylpentadecane. MAbs were precipitated from the ascitic fluid using saturated ammonium sulfate solution. Purified mAb preparations were obtained by affinity chromatography on MabTrap Kit columns (GE Healthcare Bio-Sciences, Stockholm, Sweden). Antibodies were stored at −20°C in PBS containing 0.05% NaN_3_ (pH 7.4). Monoclonal antibody isotyping was performed using the commercial Pro-Detect Rapid Antibody Isotyping Assay Kit–Mouse (Thermo Scientific). Monoclonal antibody binding constants were determined using the J method, as observed by Beatty *et al*. [17].

### HRP-conjugated polyclonal antibody preparation

Three male Chinchilla rabbits (6 months old, average body weight 3.3 kg) were subcutaneously immunized to obtain pAbs specific to recombinant *L. monocytogenes* p60 protein at multiple sites along the back. The immunization protocol lasted for 2 months with 2-week intervals between injections. On day 1, each rabbit received 1 m of antigen in 1 mL of complete Freund’s adjuvant.. On day 14, the antigen was administered in IFA, and on days 28–56 it was injected in PBS at the same dose. Blood serum was collected 3 days after the final immunization, tested for antibody production, and stored at −20°C until use.

Rabbit pAbs were conjugated with HRP using the periodate oxidation method [18]. The molar ratio of Pab to HRP was 2:1. Using the absorption ratio of 403/280 nm, a conjugation efficiency with a binding coefficient of 0.8 was achieved. For storage, an equal volume of 50% glycerol in borate buffer was added to the conjugate, which was kept at −20°C.

### Western blot

Samples of recombinant *L. monocytogenes* p60 protein (amount of protein loaded per lane: 0.25 μg, 0.5 μg, 1 μg, and 2 μg) were subjected to 10% SDS-polyacrylamide gel electrophoresis. Proteins were transferred from the gel to a polyvinylidene difluoride membrane for 50 min at 120 V. The membrane was blocked with 5% skim milk in PBS containing 0.1% Tween-20 (PBS-T) for 1 h at room temperature (RT; 20°C). After three washes with PBS-T, the membrane was incubated with MAbs from purified ascitic fluid diluted 1:100 in PBS-T for 1.5 h at +37°C. The membrane was then washed 3 times with PBS-T and incubated with HRP-conjugated anti-mouse antibodies at 1:10000 in PBS-T for 1 h at +22°C. After three washes with PBS-T and PBS, the immunoreactive bands were detected using 4-chloro-1-naphthol and captured using a scanner.

### Sandwich ELISA

Polystyrene 96-well plates were coated with 0.01 mg/mL MAbs in 0.05 M bicarbonate buffer (pH 9.6) and incubated at +4°C for 16 h. After three washes with PBS containing 0.1% Tween-20 (PBS-T) and PBS, the free surfaces of the solid phase were blocked with 1% bovine serum albumin in PBS-T for 1 h at RT. After washing, 0.1 mL of bacterial culture supernatants obtained from food isolates of *L. monocytogenes* were added to each well. To prepare the inactivated antigen, 1 mL of a 24-h *L. monocytogenes* culture grown in tryptic soy broth (TSB) was incubated at +56°C for 30 min. The culture was centrifuged at 5,500 rpm for 10 min, and the resulting supernatant was used for enzyme immunoassay.

Recombinant *L. monocytogenes* p60 antigen and TSB were used as positive and negative controls, respectively. Plates were incubated at +37°C for 1 h, followed by washing.

Next, 0.1 mL of working dilution (1:5000 in PBS-T) of HRP-conjugated rabbit pAbs raised against recombinant *L. monocytogenes* p60 antigen was added to each well and incubated at +37°C for 1 h. After washing to remove unbound components, specific antibodies were detected by enzymatic cleavage of the chromogenic substrate 3,3′,5,5′- TMB. The absorbance was measured at 450 nm using a spectrophotometer. The cutoff optical density (OD) value for positivity was calculated as the mean OD of the negative controls + 3 SDS of the negative controls.

### Isolation and identification of *L. monocytogenes* isolates

Isolation and identification of *L. monocytogenes* were performed in accordance with the requirements of ISO 11 290. Experimental studies with *L. monocytogenes* were performed under biosafety level 2 conditions, adhering to established biocontrol requirements, using personal protective equipment, working in a cabinet equipment, and standard procedures for the inactivation and disposal of biological materials.

For primary enrichment, food samples were inoculated into half-strength Fraser broth (Condalab, Madrid, Spain) at a 1:10 ratio and incubated at +30°C for 24 h. For secondary enrichment, 1 mL of the culture was transferred into full-strength Fraser broth (Condalab, Madrid, Spain) at a 1:10 ratio and incubated at +37°C for 24 h. After primary and secondary enrichment, the cultures were streaked onto CHROM agar *Listeria plates* (CHROM agar, Paris, France) to obtain single colonies and incubated at +37°C for 24–48 h. *L. monocytogenes* colonies on CHROM agar Listeria medium appear blue with an opaque halo. For confirmation, typical *L. monocytogenes* colonies were subcultured onto CHROM agar Identification *Listeria* plates (CHROM agar, Paris, France) and incubated at 37°C for 24 h. *L. monocytogenes* colonies appear pink on this chromogenic medium, surrounded by an opaque halo. To ensure accurate species determination, morphological identification on chromogenic media was further complemented by molecular and mass spectrometric approaches.

For species identification by matrix-assisted laser desorption/ionization – time-of-flight (MALDI-TOF) mass spectrometry [19], isolated colonies were grown on tryptic soy agar (HiMedia, Mumbai, India) at 37°C for 24 h. *L. monocytogenes* isolates were identified using MALDI-TOF mass spectrometry on a MALDI Biotyper Sirius System IVD (Bruker Daltonics GmbH and Co. KG, Bremen, Germany). α-Cyan-4-hydroxycinnamic acid (HCCA, Bruker Daltonics, Germany), prepared in a mixture of 50% acetonitrile and 2.5% trifluoroacetic acid, was used as a matrix. The spectra were recorded in the linear positive ion mode in the range of 2,000–20,000 m/z; the results were interpreted according to the manufacturer’s criteria.

Due to the studies, 507 animal product samples were collected. Samples for bacteriological testing were collected based on the criteria of representativeness and reliability of the microbiological assessment of the products, in accordance with ISO 11290. Samples were selected to cover a wide range of contamination sources, raw material types, and storage conditions, allowing for an objective assessment of sanitary and hygienic quality and the identification of potentially dangerous microorganisms.

*L. monocytogenes* isolation and identification were conducted in accordance with the requirements of ISO 11290. Based on the bacteriological testing results, 18 (3.55%) *L. monocytogenes* isolates were identified. Six of the 18 *L. monocytogenes* isolates, six strains were selected for the development and testing of a prototype ELISA system. The origin of the samples from various matrices (milk, meat products, and eggs) was considered. This approach allowed us to limit the number of strains to the optimal number for the primary validation of the ELISA system while maintaining the diversity of the isolates studied and ensuring the rational use of resources.

### Statistical analysis

GraphPad Prism 9.0 (GraphPad Software, USA) was used to analyze data. Results are expressed as mean ± standard deviation. Statistical significance was determined by one-way analysis of variance followed by Tukey’s *post hoc* test (p < 0.05).

## RESULTS

### Production of Recombinant L. monocytogenes p60

**Figure 1:**
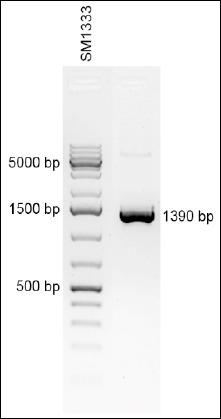
Polymerase chain reaction-amplified fragment of the *Listeria monocytogenes* *p60* gene.

The recombinant p60 protein lacking the signal peptide was selected as an antigen for generating MAbs. The N-terminal signal domain required for natural protein secretion was removed in this construct, ensuring stable expression in the heterologous *E. coli* system [20, 21]. The length of the gene encoding the target protein fragment was 1374 bp. The sequence was obtained according to the NCBI GenBank database (ID: CP023861.1, range: 618931–620304). The selected *p60* gene fragment was amplified by PCR and cloned into the pET28c(+) expression vector using NdeI and BamHI restriction sites, enabling directional in-frame insertion of the gene. The resulting construct allows the expression of the recombinant protein with an N-terminal hexahistidine tag (6× His), facilitating subsequent purification by affinity chromatography. Figure 1 shows the amplified gene fragment.

**Figure 2:**
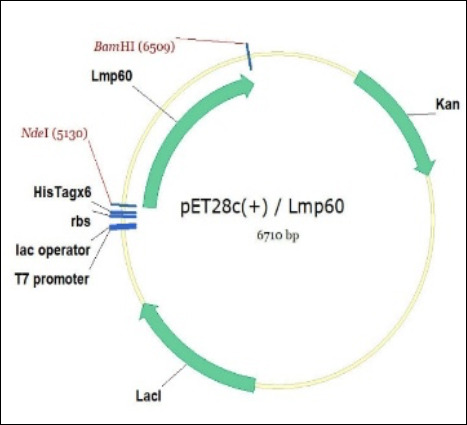
Genetic map of the pET28c(+)/Lmp60 plasmid.

The PCR product is 1390 bp in length, comprising a 1374 bp fragment of the *p60* gene and 16 bp of additional sequences carrying the NdeI and BamHI restriction sites. The *p60* gene was cloned into the pET28c(+)/Lmp60 expression plasmid under the control of the T7 promoter. The plasmid map of the construct is shown in Figure 2.

**Figure 3:**
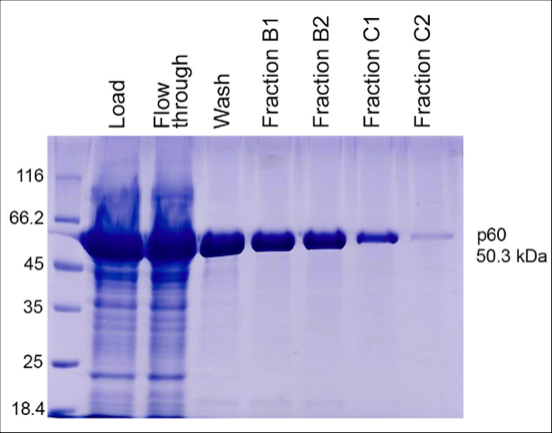
Purification of the recombinant *Listeria monocytogenes* p60 protein.

The design provides for the expression of the recombinant p60 protein fragment fused with an N-terminal 6× His tag for affinity purification. The open reading frame encodes a protein of 478 amino acids with a calculated molecular mass of approximately 50.3 kDa. Recombinant p60 was purified by affinity chromatography using a HisTrap HP column (Cytiva) with immobilized Ni^2+^ ions. Elution was performed under a linear imidazole gradient ranging from 50 mM to 500 mM. A highly purified recombinant p60 protein was obtained, with a yield of 5.9 mg/L (Figure 3).

The recombinant p60 protein was then dialyzed against PBS (pH 7.4) to remove residual imidazole and other low-molecular-weight components. Dialysis was performed at 4°C with multiple buffer exchanges. The purified and dialyzed protein was subsequently used as an antigen for immunizing experimental animals.

### Immunization of BALB/c mice and hybridoma production

Inbred BALB/c mice were immunized with recombinant *L. monocytogenes* p60 protein to obtain hybridoma strains producing MAbs.

A 2-week immunization scheme in BALB/c mice was effective in stimulating the immune system to induce B lymphocyte clones that produced high levels of antigen-specific antibodies (Table 1). Five independent hybridizations of X63–Ag 8.6.5.3 myeloma cells with immune splenocytes from BALB/c mice were performed to achieve optimal results.

**Table 1:** Induction of B-lymphocyte clones in the spleens of mice immunized with BALB/c.

Immunogen	Antigen dose	Mean antibody titer (M ± m) (n = 3)	Mean number of splenic B lymphocytes in immunized mice (10^6^×, n = 3)
Recombinant p60 protein	25 μg protein	1:10666 ± 2.133	57.3 ± 4.7
	50 μg protein	1:21333 ± 4.266	83.3 ± 9.1

**Figure 4:**
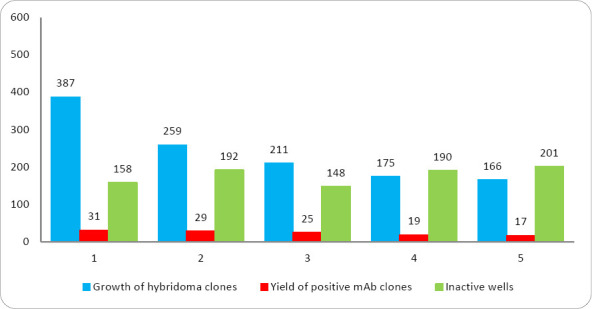
Hybridoma growth after myeloma cell fusion with splenocytes from **BALB/c** mice immunized with recombinant *Listeria monocytogenes* p60 protein.

Robust hybridoma growth was observed in 166–387 wells out of 384–576 seeded, corresponding to a high cell fusion efficiency of 43.2%–67.1%. Primary screening of culture supernatants from hybridomas revealed antibody activity against recombinant p60 in 121 clones out of 1198 hybridomas, accounting for 10.1% (Figure 4).

**Figure 5:**
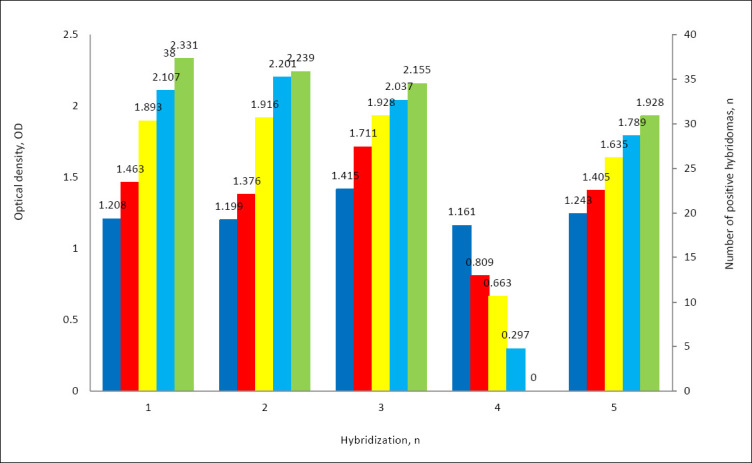
Yield of positive hybridoma clones based on five rounds of screening.

During the fourth round of screening, 94.2% of the hybridoma clones lost the ability to bind the target antigen or exhibited low titers (1:2 or 1:4) (Figure 5). Stable secretion of specific immunoglobulins was observed in only 7 hybridoma clones (1H8, 2A3, 2G4, 5F9, 5D2, 6B9, and 7E7), with maximum culture supernatant titers ranging from 1:16 to 1:256.

### Immunochemical characterization of MAbs

*In vivo* cultivation of hybridoma cells resulted in a significant increase in the concentration of MAbs (after purification on a MabTrap [Thermo Fisher Scientific] column) in ascites fluid, up to 8–16 mg/mL. Monoclonal antibody isotyping revealed that clone 1H8 antibodies are immunoglobulin G (IgG)1 immunoglobulins, while clones 2A3 and 7E7 were identified as IgG2a and IgG2b, respectively.

The specific activity of hybridoma strains 1H8, 2A3, and 7E7 producing MAbs was determined by indirect ELISA (Table 2). According to the ELISA specificity results, MAbs 1H8 and 2A3 demonstrated the highest specificity to recombinant p60 protein of *L. monocytogenes*, with titers of 1:256 in culture supernatant and 1:204,800 in ascitic fluid. In contrast, mAb 7E7 showed lower binding levels, with titers of 1:128 and 1:102,400. The specificity of MAbs 1H8, 2A3, and 7E7 was further confirmed by the absence of cross-reactivity with recombinant antigens from related microorganisms, Lip32 of *Leptospira hebdomadis* and BP26 of *Brucella abortus*.

**Table 2:** Specificity and activity of monoclonal antibodies from hybridoma strains in indirect enzyme-linked immunosorbent assay.

Antigens	1H8	2A3	7E7
Recombinant antigens			
p60 *Listeria monocytogenes*	1:256 / 1:204800	1:256 / 1:204800	1:128 / 1:102400
Lip 32 *Leptospira hebdomadis*	NR	NR	NR
BP26: *Brucella abortus*	NR	NR	NR
Suspension of the bacterial cells			
*Listeria monocytogenes*	1:128 / 1:102400	1:64 / 1:51200	1:32 / 1:25600
*Escherichia coli*	NR	NR	NR
*Salmonella Typhimurium*	NR	NR	NR
*Staphylococcus aureus*	NR	NR	NR

Numerator = Culture supernatant, Denominator = Ascitic fluid, NR = Negative reaction

The tested mAb types demonstrated high-affinity for intact *L. monocytogenes* cell suspensions at titers of 1:25,600–1:102,400, confirming the surface localization of the detectable outer membrane protein p60 (murein hydrolase). The specificity of *L. monocytogenes* p60 antigen detection by the tested MAbs was further demonstrated by the absence of binding to intact cells of heterologous microorganisms, *E. coli*, *Salmonella Typhimurium*, and *Staphylococcus aureus*, in both culture and ascites fluid.

**Figure 6:**
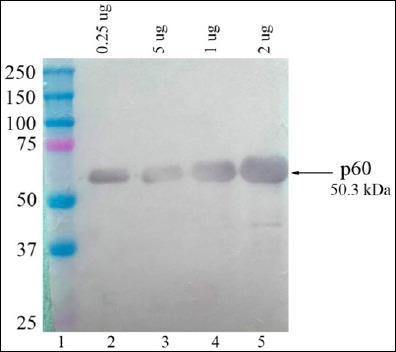
Western blot analysis of recombinant p60 antigen probed with monoclonal antibodies from hybridoma strain 1H8: Lane 1: Molecular weight marker (#1610374, Bio-Rad); lanes 2–5; p60 protein (0.25 μg, 0.5 μg, 1 μg, and 2 μg).

Western blotting of recombinant p60 protein with MAbs from hybridoma strain 1H8 confirmed the specificity of antibody recognition to the *L. monocytogenes* p60 protein target epitope (Figure 6). The immunoblot revealed an immunopositive protein fraction with a molecular mass of approximately 50.3 kDa, consistent with the expected molecular weight of the recombinant p60 antigen.

### Development and evaluation of the ELISA test system

The highly sensitive immunodetection of pathogenic *Listeria* in food products was performed using the “sandwich” ELISA format. This format relies on the formation of an immunocomplex consisting of immobilized MAbs (capture MAbs) + *L. monocytogenes* p60 protein + enzyme-labeled pAbs (detection pAbs). The sandwich format provides superior sensitivity compared to alternative assay designs, such as competitive or indirect ELISA.

To obtain the second immunological component of the test system, rabbits were immunized with pAbs specific to recombinant *L. monocytogenes* p60 protein.

**Figure 7:**
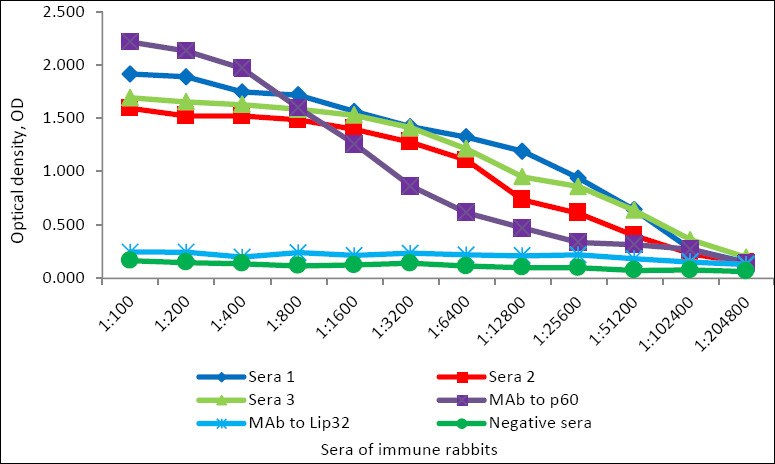
Titers of specific antibodies in the sera of rabbits immunized with recombinant *Listeria monocytogenes* p60 protein (n = 3).

The prolonged immunization schedule in rabbits with the recombinant p60 protein resulted in a high level of specific antibody production against the target antigen. The mean maximum antibody titer in the sera of immunized rabbits reached 1:170,666 (n = 3), whereas sera from control (non-immunized) rabbits showed no reactivity (Figure 7). The optimal working dilution of HRP-conjugated rabbit pAbs, prepared by the periodate oxidation method, was determined to be 1:5000. This dilution provided minimal background absorbance (OD = 0.072) and maximum signal intensity (OD = 1.812).

For the sandwich ELISA, hybridoma-derived monoclonal antibody 1H8, specific for recombinant p60 protein, was selected as the capture antibody. The immobilization of mAb 1H8 at 5 µg/mL completely saturated the active binding sites of the polystyrene surface, as evidenced by the maximal OD values for the positive control (OD 1.868–2.004) and minimal OD for the negative control (OD 0.052–0.058). Both lower (2.5 µg/mL) and higher (10 µg/mL) coating concentrations resulted in a 30%–35% reduction in signal intensity and increased background staining.

**Figure 8:**
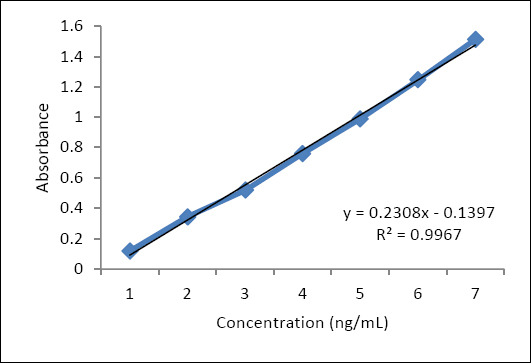
Analytical sensitivity of the sandwich enzyme-linked immunosorbent assay test system.

The analytical sensitivity of the sandwich ELISA was evaluated using twofold serial dilutions of recombinant *L. monocytogenes* p60 protein in buffer at concentrations ranging from 1 µg/mL to 1 ng/mL. Based on capture MAbs and enzyme-labeled pAbs specific for recombinant p60, the minimum detection limit of the assay was 1.5 ng/mL (Figure 8). The high sensitivity of the sandwich ELISA can be attributed to the strong binding affinity of the capture mAb (association constant, Ka = 2.5 × 10^10^ M^1^), which confers excellent antigen-binding activity.

### Diagnostic evaluation using field isolates

*L. monocytogenes* isolates obtained from food products were used to evaluate the diagnostic efficiency of the ELISA test system (Table 3). The identification of pathogenic *Listeria* in food samples was confirmed by bacteriological analysis, including two-step enrichment in selective Fraser broth, subculturing on CHROMagar Listeria, microscopy, and catalase testing. Identification was also performed using chromogenic media CHROMagar Listeria and CHROMagar Identification Listeria (CHROMagar, France). *L. monocytogenes* colonies exhibited characteristic blue and pink coloration with an opaque halo on these media.

**Table 3:** Isolates of *Listeria monocytogenes* recovered from food products (n = 6).

Genus	Species	Identification code	Source
*Listeria*	*Monocytogenes*	368–1	Dumplings
*Listeria*	*Monocytogenes*	457	Beef
*Listeria*	*Monocytogenes*	458	Pork
*Listeria*	*Monocytogenes*	636–1	Chicken meat
*Listeria*	*Monocytogenes*	721–1	Dumplings
*Listeria*	*Monocytogenes*	795	Milk

Species-level identification was confirmed using MALDI-TOF mass spectrometry. In all *L. monocytogenes* isolates (C1–C6), the score values ranged between 1.800 and 2.000, which corresponds to a high probability of species-level identification. These results corroborate the preliminary microbiological and biochemical data and provide a reliable pathogen identity confirmation (Table 4).

**Table 4:** Comparative evaluation of the performance of the developed ELISA test system with commercial kits.

Parameters	Listeria detection system		

	Developed an ELISA test system	“Solus Listeria ELISA”	“Listeria p60 ELISA Kit” (Cell Biolabs, USA)
Target antigen	p60 (Kazakhstani isolate)	p60 (reference strain)	p60 (reference strain)
Capture antibodies	Mouse Mab (intellectual property)	Mouse Mab (intellectual property)	Mouse Mab (intellectual property)
Detection antibodies	Rabbit Pab	Mouse Mab	Mouse Mab
Detection limit	1,5 ng/mL	1,5 ng/mL	625 pg/mL
Assay duration	2 h	3 h	4 h
Cross-reactivity	None	None	None
Kit cost	15$	420$	385$

ELISA = Enzyme-linked immunosorbent assay

**Figure 9:**
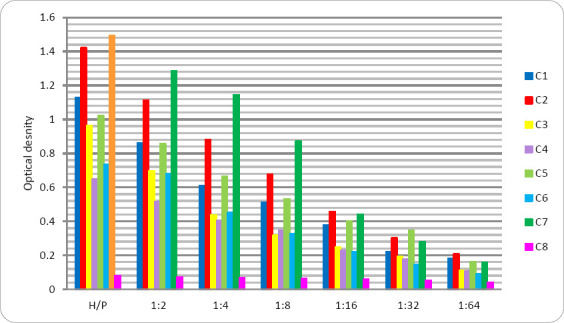
Detection of p60 in culture supernatants of *Listeria monocytogenes* strains isolates C1-C6 (n = 6); standard p60 protein (C7), and tryptic soy broth (C8).

The detection of the p60 protein of pathogenic *Listeria* strains was performed using the developed ELISA test system in the culture supernatants of 24-h *L. monocytogenes* suspensions isolated from food products. The ELISA test system yielded positive results for the specific detection of *L. monocytogenes* p60 antigen in the culture supernatants of all food isolates (Figure 9).

**Figure 10:**
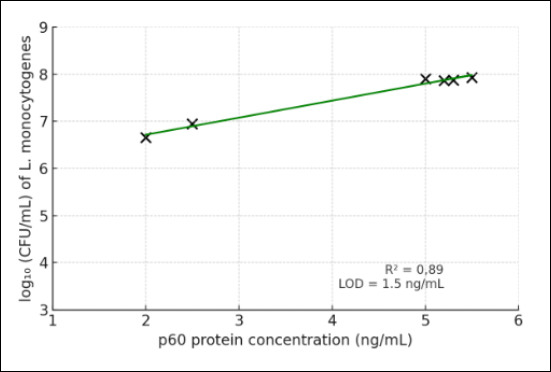
Calibration curve reflecting the quantitative relationship between p60 protein concentration (ng/mL) and the logarithm of *Listeria monocytogenes* viable cell count (colony-forming unit/mL).

A linear relationship (R^2^ = 0.89) was established between the p60 protein concentration and the viable cell count of *L. monocytogenes*, expressed as log_10_ CFU/mL (Figure 10). The developed ELISA system demonstrated reliable analytical sensitivity in the range of 1.5 ng/mL, with a detection limit corresponding to approximately 10^3^ CFU/mL. These results confirm the feasibility of quantitatively determining bacterial equivalents based on antigen concentration in food matrices.

The analytical efficiency and reduced assay time demonstrate that the developed ELISA is both sensitive and regionally adapted, representing a new alternative to imported diagnostic kits (Table 4).

The developed immunoassay enables the rapid identification of pathogenic *Listeria* strains, significantly reducing the time required for analysis while increasing detection reliability, even at low pathogen concentrations in the tested samples.

## DISCUSSION

### Importance of rapid detection of *L. monocytogenes*

*L. monocytogenes* is a potential contaminant of raw materials and food products, which are the primary transmission vehicles and play a major role in the occurrence of human listeriosis [1–3]. The rapid detection and reliable identification of *L. monocytogenes* antigens in food samples or environmental sources are essential for implementing effective control strategies to prevent listeriosis outbreaks.

### Diagnostic relevance of the p60 protein

During bacterial cell division, the p60 protein is secreted in large quantities (up to 75%) into the extracellular environment, making it a highly valuable diagnostic marker and an ideal indicator protein for the detection of *L. monocytogenes* in contaminated food products [13–16]. Structural studies have demonstrated that the three-dimensional organization of *L. monocytogenes* p60 is domain-based, with conserved regions at the N- and C-termini. The protein’s central part is highly variable among *Listeria* species. The C-terminal region has been identified as the catalytic domain, whereas the N-terminal region functions in substrate (peptidoglycan) recognition and binding. In the conserved N-terminal region, amino acid residues 1–27 encode a signal peptide sequence [4–6, 11, 22].

### Role of antibodies in p60-based immunodiagnostics

Both pAbs and MAbs raised against key immunodominant antigens of *L. monocytogenes* have proven useful as specific immunoreagents for the development and optimization of ELISA-based methods for listeriosis surveillance and prevention. The use of either full-length or peptide fragments of the p60 protein is effective for generating highly specific MAbs and designing immunoassays capable of detecting pathogenic *Listeria* in food products.

### Existing p60-targeted MAbs and assay systems

The successful development and application of MAbs directed against the full-length *L. monocytogenes* p60 protein in sandwich ELISA for the detection of *Listeria* in food products were previously described by Yu *et al*. [15]. Among these, mAb p6007 specifically recognized p60 in different *L. monocytogenes* serotypes, whereas mAb p6017 exhibited broader reactivity with p60 across *Listeria* species, highlighting differences in epitope specificity. Similarly, Lim *et al*. [23] demonstrated that the hybridoma-derived mAb 1H4 recognized all members of the genus *Listeria* without showing cross-reactivity with closely related microorganisms. The high-specificity of MAbs 10E7 and 9H3 against full-length *L. monocytogenes* p60 enabled their application in gold nanoparticle-based immunochromatographic assays, achieving detection limits of 10^3^–10^4^ CFU/mL [24].

Selective detection of *L. monocytogenes* was also achieved using MAbs directed against a short, highly conserved peptide within the variable central region of the p60 protein. The hydrophilic 11-amino-acid peptide PepD (QQQTAPKAPTE) was identified as a conserved motif unique to *L. monocytogenes* serotypes. Expression of this unique fragment allowed the generation of highly specific antibodies capable of recognizing various *L. monocytogenes* serotypes [13]. This peptide was effectively employed as a target for developing an immunofluorescence-based assay for the detection of *Listeria* in food products [16]. Application of MAbs with similar epitope specificity in sandwich ELISA enabled assay sensitivity as low as 10^3^ CFU/mL [14].

### Rationale for using a regional p60 antigen in this study

Unlike previous ELISAs developed using standard reference strains, this study utilized a recombinant p60 antigen obtained from a locally circulating *L. monocytogenes* isolate in Kazakhstan. The antigenic match of the resulting MAbs to the regional *Listeria* field strain significantly increases the diagnostic value of regionally contaminated food product detection. The development of a fully functional ELISA test system based on domestically produced immunoreagents will reduce reliance on imports.

### Structural domains and immunological relevance of p60

Bioinformatic analyses of the structural organization of p60 revealed that the N-terminal region of the full-length protein contains two Lysin motif domains separated by an SH3 domain, whereas the NlpC/p60 domain is present in the C-terminal region [25]. Functional studies of these structural domains have shown that the N-terminal LysM domain plays primary immunomodulatory and immunostimulatory roles [19, 25]. This small protein module, consisting of approximately 50 amino acids, was identified as a major immune-binding site with high-affinity for N-acetylglucosamine polymers in peptidoglycan [19, 25, 26]. Thus, the secretion of p60 into the host cytosol is accompanied by the cleavage of its signal peptide, thereby exposing and activating the immunologically relevant N-terminal domains [20].

### Advantages of expressing p60 without the signal peptide

Thus, a fragment of the p60 protein gene lacking the N-terminal signal peptide was strategically expressed to increase protein solubility, prevent inclusion formation, and preserve the immunogenic LysM domains responsible for host interaction. This rational design increases antigen yield, stability, and accessibility of diagnostic epitope features not present in previous ELISA systems for *Listeria*. Using the *p60* gene, which lacks the N-terminal signal domain, from the genomic DNA of the regional isolate *L. monocytogenes*, we constructed a genetic system to produce the recombinant antigen and demonstrated the accumulation of recombinant p60 protein in induced cultures of the expression strain *E. coli* arctic express (DE3)/pET28c(+)/Lmp60. Note that the use of the arctic express (DE3) producer strain to express soluble p60 protein under low-temperature induction conditions is rare in the case of *Listeria* antigens.

### Immunogenicity and hybridoma production efficiency

The use of this immunologically active form of p60 lacking the signal peptide stimulated a strong immune response in experimental animals, resulting in the generation of sufficient B lymphocytes producing specific antibodies. The success of obtaining hybridomas producing MAbs is largely determined by the efficiency of immunization of Balb/c mice, which serve as donors of immune lymphocytes for fusion with myeloma cells [7, 27]. The yield of positive clones among all hybridomas reflected the strong immune background of the B-cell pool and the feasibility of isolating active clones with desired properties.

### Selection of high-performance MAbs

The primary antibody is a key immunoreagent that defines the immunoassay specificity [28]. Based on its high binding affinity (Ka = 2.5 × 10^10^ M^-1^) and low background signal from seven stable hybridoma lines producing MAbs to the recombinant p60 antigen, the mAb clone 1H8 was used to develop the assay. The use of immunoreagents of different species (mouse capture mAb 1H8 + rabbit detection pAb-HRP) for *Listeria* p60 detection in a sandwich ELISA significantly reduced nonspecific interactions. The specific antigen targeting of the mAb-pAb antibody pair against the same recombinant antigen ensures precise epitope recognition and increases the assay accuracy.

Using hybridoma technology, we obtained stable hybridoma cell lines (1F9, 1E7, and 6C12) that produced high-specificity MAbs against *L. monocytogenes* recombinant p60. Immunochemical characterization of these antibodies by ELISA and immunoblotting confirmed their high activity and specificity. Since p60 is secreted in large amounts during the active growth of pathogenic *Listeria*, it represents a more advantageous diagnostic target than other antigens [13–16, 29].

### Development of the sandwich ELISA and its analytical performance

In this study, we developed a sandwich ELISA using high-affinity murine MAbs against the p60 protein of *L. monocytogenes* as the primary capture antibodies, together with rabbit pAbs of the same specificity conjugated with HRP as the secondary detection antibodies. The assay demonstrated analytical sensitivity (0.5–0.75 ng/mL) comparable to that of widely used commercial kits, such as the “Solus Listeria ELISA Kit” (PerkinElmer, USA) and the “*Listeria monocytogenes* p60 ELISA Kit” (Cell Biolabs, USA). Given the genetic divergence of *Listeria* strains from different geographic regions, the use of a Kazakhstani field isolate to produce a recombinant p60 antigen provides increased diagnostic sensitivity for the detection of regionally prevalent *Listeria* genotypes.

### Advantages of domestic immunoreagent production

Domestically produced immunoreagents for ELISA test systems will be cost-effectively manufactured using existing regional infrastructure, providing an accessible diagnostic platform for national food safety surveillance programs. This also contributes to the WHO’s One Health goal of strengthening zoonotic pathogen monitoring.

### Validation using regional *L. monocytogenes* isolates

The method was successfully validated for detecting p60 in the supernatants of bacterial suspensions of six *L. monocytogenes* strains isolated from food products. The results demonstrated that the developed sandwich ELISA enables direct and specific analysis of bacterial culture suspensions and is suitable for the detection of *Listeria* in food samples. This platform creates a scalable immunodiagnostic technology that integrates the development of recombinant antigens, the creation of mAb hybridomas, and the assembly of test systems into a single, reproducible workflow, paving the way for the development of immunoassays for other zoonotic pathogens.

## CONCLUSION

This study successfully developed and validated a regionally adapted sandwich ELISA for the rapid and sensitive detection of *L. monocytogenes* using a recombinant p60 antigen derived from a locally circulating Kazakhstani field isolate. The recombinant antigen lacking the N-terminal signal peptide demonstrated high solubility and strong immunogenicity, enabling the production of high-affinity MAbs (notably clone 1H8, Ka = 2.5 × 10^10^ M^-1^) and potent HRP-conjugated rabbit pAbs. The optimized assay achieved an analytical sensitivity of 1.5 ng/mL, corresponding to approximately 10^3^ CFU/mL, and yielded consistent positive detection across all tested *L. monocytogenes* isolates from food samples. The strong linear correlation (R^2^ = 0.89) between antigen concentration and bacterial load further underscores the reliability of the assay for quantitative detection.

The ELISA platform offers a rapid, affordable, and technically accessible diagnostic tool suitable for food industries, public health laboratories, and national surveillance programs. By enabling early detection of *L. monocytogenes* in raw materials and processed foods, the assay supports timely intervention and reduces the risk of listeriosis outbreaks. The use of domestically produced immunoreagents reduces dependence on imported kits and enhances diagnostic responsiveness in resource-limited or region-specific settings.

Key strengths of this study include: (i) the use of a regionally relevant antigen that improves diagnostic match to circulating strains, (ii) high-affinity MAbs with excellent specificity and no cross-reactivity with heterologous bacteria, (iii) reproducible hybridoma lines enabling sustainable reagent production, and (iv) a complete antigen-to-assay development pipeline adaptable for other pathogens.

Some limitations include the limited number of field isolates used for validation, reliance on culture supernatants rather than complex food matrices, and the need for broader multicenter evaluation across additional geographic regions and food products. Furthermore, matrix effects and potential inhibitors in processed foods were not examined and require future consideration.

Expanding the assay to incorporate pre-enrichment steps, developing a rapid lateral-flow version for on-site detection, evaluating cross-regional strain diversity, and integrating quantitative algorithms for antigen–load correlation represent important future directions. In addition, the modular design of this platform can be adapted for the detection of other zoonotic pathogens by substituting antigen–antibody pairs.

Overall, this study establishes a highly sensitive, specific, and regionally optimized ELISA for the detection of *L. monocytogenes*, providing a significant advancement in food safety diagnostics. By integrating recombinant antigen engineering, hybridoma technology, and optimized assay design, the platform strengthens One Health–aligned surveillance efforts and provides a scalable foundation for developing next-generation immunodiagnostic tools.

## DATA AVAILABILITY

All the generated data are included in the manuscript.

## AUTHORS’ CONTRIBUTIONS

GK and GU: Hybridoma technology, optimization of test system parameters. SM and ZA: Development of test system and determination of diagnostic efficiency. AT and SA: Gene synthesis, cloning, expression, and purification of recombinant antigen. PS, AG, and RR: Isolation and identification of isolates, MALDI-TOF, and preparation of bacterial suspension. AR and YA: Result interpretation and drafted the manuscript. SE: Project design and supervision. All authors have read and approved the final version of the manuscript.
